# The Gut Microbiota–Immunity Axis in Colorectal Cancer: Implications for Immunotherapy and Clinical Translation

**DOI:** 10.3390/cells15191800

**Published:** 2026-10-01

**Authors:** Izabela Siemińska

**Affiliations:** 1 Department of Clinical Immunology, Institute of Paediatrics, Jagiellonian University Medical College, Wielicka 265, 30-663 Krakow, Poland; i.gorska@uj.edu.pl or izabela.sieminska@urk.edu.pl; 2 Department of Infectious Diseases and Public Health, Faculty of Veterinary Medicine, University of Agriculture in Cracow, 30-059 Krakow, Poland

**Keywords:** colorectal cancer, fecal microbiota transplantation, gut microbiota, immune checkpoint inhibitors, tumor immune microenvironment

## Abstract

Colorectal cancer (CRC) arises within a complex microbial ecosystem that may influence carcinogenesis, tumor immunity, and therapeutic response. This is particularly relevant in metastatic disease, where most tumors are microsatellite-stable/mismatch repair-proficient (MSS/pMMR) and derive little meaningful benefit from immune checkpoint inhibitor (ICI) monotherapy. Specific pathobionts, including *Fusobacterium nucleatum*, enterotoxigenic *Bacteroides fragilis*, and colibactin-producing *Escherichia coli*, have been implicated in genotoxic, inflammatory, and checkpoint-related mechanisms that may contribute to tumor progression and immune evasion, whereas selected commensals and microbiota-derived metabolites may support antitumor immunity in a context-dependent manner. This review integrates translational evidence linking microbial taxa and functions, metabolites, neutrophil biology, bacterial extracellular vesicles, and T-cell dysfunction with CRC progression and immunotherapy response. This review critically examines emerging microbiome-directed strategies, including fecal microbiota transplantation, next-generation probiotics, defined bacterial consortia, dietary and perioperative interventions, postbiotics, and bacteriophage-based approaches, as potential means of modifying immune resistance in MSS/pMMR CRC. Although early clinical signals from microbiota-modulating combinations are encouraging, the available evidence remains limited by small cohorts, heterogeneous interventions, and the absence of randomized CRC-specific studies. Microbiome-based biomarkers and therapeutics therefore remain investigational, and standardized sampling, prospective validation, and carefully designed trials will be required before microbiome modulation enters routine CRC care.

## 1. Introduction

Colorectal cancer (CRC) is the third most commonly diagnosed malignancy and the second leading cause of cancer-related mortality worldwide, with approximately 1.9 million new cases and 900,000 deaths annually [1]. The human colon harbors the highest microbial density in the body, and this proximity has direct consequences for tumor biology: commensal bacteria support the mucosal immune system under physiological conditions, while the shift toward a dysbiotic community—enriched in oncogenic pathobionts and depleted of short-chain fatty acid (SCFA)-producing species—promotes chronic inflammation, epithelial barrier breakdown, and active suppression of antitumor immunity [2].

Approximately 85% of all CRCs and about 95% of metastatic cases are MSS/pMMR [2,3]. In contrast to dMMR/MSI-H tumors, which typically have a higher mutational and neoantigen burden and are more responsive to immune checkpoint inhibitors (ICIs), MSS/pMMR tumors generally show lower immunogenicity, limited T-cell infiltration, impaired antigen presentation, and an immunosuppressive tumor microenvironment [2,3]. Within this multifactorial landscape, the gut microbiota has emerged as one of several potentially modifiable factors that may influence systemic and intratumoral immunity and modulate responses to ICIs [4]. Fecal microbiota transplantation (FMT), next-generation probiotics (NGPs), and dietary interventions are consequently being investigated as adjunctive microbiome-modulating strategies, although their ability to overcome ICI resistance in MSS/pMMR CRC remains unproven [4].

This review synthesizes the mechanistic and translational evidence with a deliberate clinical emphasis: what each microbial mechanism means for patient care, where the evidence is strong enough to guide practice, and where it remains preliminary. It covers the dysbiotic changes that characterize CRC, how they suppress antitumor immunity, the clinical data on microbiome-directed sensitization to ICIs, and the current state of therapeutic microbiome modulation, from perioperative probiotics supported by randomized controlled trial (RCT) data to experimental combinations of FMT with immunotherapy in MSS mCRC. Particular emphasis is placed on clinically relevant, mechanistically defined microbial functions, including secondary bile acid metabolism, bacterial extracellular vesicles, and neutrophil biology, while distinguishing CRC-specific evidence from findings extrapolated from other tumor types or preclinical models.

## 2. Microbiota in Colorectal Carcinogenesis

CRC-associated microbiomes differ from those of healthy individuals, with changes in microbial diversity and composition, depletion of several SCFA-producing taxa, and enrichment of selected pathobionts [5]. Similar alterations have been detected in premalignant adenomas, suggesting that dysbiosis emerges early in colorectal carcinogenesis, although its causal contribution is unresolved [6]. Three bacterial groups have attracted particular attention because of their mechanistic and potential clinical relevance.

*Fusobacterium nucleatum* is one of the best-characterized tumor-associated bacteria in CRC. Higher fecal or intratumoral abundance has been associated with poorer overall and disease-free survival, postoperative recurrence, lymph-node metastasis, and reduced response to chemotherapy [7,8]. These findings vary between cohorts but collectively link *F. nucleatum* to a more aggressive disease phenotype.

Beyond its immunomodulatory effects, *F. nucleatum* may promote CRC progression through FadA-mediated binding to epithelial E-cadherin and activation of β-catenin signaling, as well as through inflammatory signaling; these mechanisms have been demonstrated predominantly in experimental models. Preclinical studies also implicate *F. nucleatum* in resistance to immunotherapy, although direct evidence from patients with CRC treated with ICIs is limited. Mechanistically, the bacterium can suppress the activity of tumor-infiltrating T cells and natural killer cells by engaging inhibitory receptors, particularly TIGIT, and can promote the accumulation of immunosuppressive myeloid cells [8,9,10]. It may also increase PD-L1 expression in CRC cells, providing a potential link between bacterial signaling and immune-checkpoint regulation [11].

These observations support investigating bacterium-directed interventions alongside anti-PD-1 or anti-TIGIT therapy. Whether such combinations can improve clinical outcomes in CRC remains to be determined.

Another well-characterized CRC-associated microorganism is enterotoxigenic *Bacteroides fragilis* (ETBF), a toxin-producing strain of *B. fragilis*. Its secreted toxin, *B. fragilis* toxin (BFT; fragilysin), disrupts epithelial integrity, in part through cleavage of E-cadherin, and activates IL-17- and STAT3-dependent inflammatory signaling [12,13]. The resulting chronic inflammation can promote tumor growth while limiting effective antitumor immunity. ETBF is detected more frequently in CRC tissue than in non-neoplastic controls and has been reported to co-occur with *F. nucleatum* [14]. Whether this reflects functional cooperation between the two bacteria, rather than their shared preference for the tumor-associated niche, remains uncertain.

pks^+^ *Escherichia coli* acts through a different mechanism. It produces colibactin, a genotoxin that damages DNA in colonic epithelial cells and leaves a traceable mutational fingerprint, represented by the SBS88 and ID18 signatures, in human CRC genomes [14]. These signatures are more frequent in early-onset CRC, providing strong molecular evidence that exposure to colibactin-producing bacteria may contribute to colorectal carcinogenesis [14]. Beyond its genotoxic effects, pks^+^ *E. coli* can remodel the tumor microenvironment through altered lipid metabolism, reduced CD8^+^ T-cell infiltration and increased survival of cancer cells exposed to chemotherapy [15].

The loss of potentially beneficial commensals may also contribute to CRC-associated dysbiosis. Several studies have reported depletion of SCFA-producing bacteria, including *Faecalibacterium prausnitzii* and selected members of the Ruminococcaceae family. Butyrate, produced through microbial fermentation of dietary fiber, is an important energy source for colonocytes and contributes to epithelial integrity and immune homeostasis. It can inhibit pro-inflammatory NF-κB signaling and, at higher local concentrations, exert antiproliferative and pro-apoptotic effects in CRC cells [16]. Reduced butyrate production may therefore weaken epithelial and immune mechanisms that normally restrain inflammation and tumor development.

*Akkermansia muciniphila* supports intestinal barrier integrity and modulates dendritic-cell activation, but its role in CRC appears context-dependent. Studies examining its abundance across colorectal carcinogenesis have produced inconsistent results, with some reporting a decline during disease progression and others finding enrichment in established CRC or context-specific tumor-promoting effects [17,18]. Consequently, endogenous *A. muciniphila* abundance cannot currently be interpreted as a simple marker of disease stage or prognosis. *Bifidobacterium* species support antigen-presenting cell function and CD8^+^ T-cell priming, and their depletion is associated with reduced ICI response across multiple tumor types [19].

## 3. Microbial Modulation of the Tumor Immune Microenvironment

Dysbiosis may reinforce immunosuppression within the colorectal cancer tumor immune microenvironment (TIME). Microbial signals can impair cytotoxic T-lymphocyte and natural killer (NK)-cell activity, disrupt antigen presentation by dendritic cells, promote the accumulation of immunosuppressive myeloid populations, and alter immune-checkpoint expression. These effects contribute to, but do not fully explain, the immunologically “cold” phenotype of most MSS tumors and their limited responsiveness to immune checkpoint inhibitor monotherapy [2,20]. The microbiota can influence this environment locally, through bacteria and bacterial products entering tumor tissue, and systemically, through microbial metabolites reaching the tumor via the circulation.

Beyond direct local signaling and circulating metabolites, microbial cues may also induce more persistent metabolic and epigenetic reprogramming of immune cells, although the relevance of these mechanisms in human CRC remains incompletely defined.

SCFAs, produced through microbial fermentation of dietary fiber, are important modulators of the TIME, but their effects vary with the metabolite, concentration, target cell, and tissue context. Butyrate can promote regulatory T-cell differentiation and IL-10 production, thereby supporting immune tolerance. Under different conditions, however, it can enhance CD8^+^ T-cell effector and memory responses through mechanisms that include histone deacetylase inhibition and metabolic reprogramming [21,22,23]. In preclinical models, these effects can increase cytokine production and the antitumor activity of cytotoxic T cells [21,22]. Clinical studies, conducted predominantly in melanoma and other solid tumors, have also associated higher systemic SCFA concentrations with improved responses to anti-PD-1 therapy [23]. The immunological activity of SCFAs therefore cannot be reduced to a simple low-versus-high concentration model, but depends on the specific metabolite, immune-cell subset, receptor expression, tissue environment, and disease context. In murine models of CRC, *Lactobacillus plantarum* L168-derived indole-3-lactic acid increased H3K27 acetylation at Il12a enhancer regions in dendritic cells, thereby enhancing IL-12 production and CD8^+^ T-cell priming; however, the clinical relevance of this mechanism in CRC remains to be established [24].

This context dependence is illustrated by an intestinal tumor-on-chip model in which physiological concentrations of butyrate and propionate reduced the cytotoxicity of chimeric antigen receptor (CAR) T cells targeting a colorectal tumor antigen [25]. Although this observation cannot be directly extrapolated to patients, it shows that increasing SCFA availability may not enhance every form of cancer immunotherapy. The relevant metabolite, concentration, immune effector cell, and therapeutic modality must all be considered.

Microbial bile acid metabolism provides another link between the gut microbiota and antitumor immunity. In a 2024 study, Cong et al. identified a potentially targetable mechanism involving deoxycholic acid (DCA), a secondary bile acid generated through bacterial conversion of primary bile acids [26]. DCA inhibited calcium signaling in CD8^+^ T cells and reduced their production of granzyme B, interferon-γ, and tumor necrosis factor-α, thereby limiting their cytotoxic activity. In patients with CRC, higher intratumoral DCA concentrations correlated negatively with CD8^+^ T-cell effector function. Targeted depletion of DCA-producing bacteria using bacteriophages or pharmacological sequestration of bile acids restored CD8^+^ T-cell activity and suppressed tumor growth in mice [26].These findings identify microbial DCA metabolism as a defined immunosuppressive mechanism and a potential therapeutic target in CRC.

The effects of secondary bile acids are nevertheless not uniform. Lithocholic acid and its derivatives can influence regulatory T cells and immunosuppressive myeloid populations, whereas other bile acids, including ursodeoxycholic acid and taurolithocholic acid, may exert anti-inflammatory or immunoregulatory effects in specific experimental settings [27]. Their biological activity depends on the receptor involved, target cell, metabolite concentration, and local tissue environment.

Neutrophils provide another link between the microbiota and the immune environment of CRC. *F. nucleatum* can activate neutrophils and promote the formation of neutrophil extracellular traps (NETs). Although NETs have been associated with metastatic progression, the proposed shielding of tumor cells from cytotoxic lymphocytes remains largely inferential and is supported mainly by preclinical evidence [28,29]. The microbiota may additionally shape neutrophil function through systemic signals. Germ-free mice exhibit altered neutrophil aging programs, illustrating that microbial exposure can influence neutrophil phenotype even beyond the intestine [30]. In a recent study of CRC liver metastasis, bile salt hydrolase-deficient microbiota increased conjugated primary bile acids; taurocholic acid then promoted TIMP1-dependent CXCL5/CXCR2-mediated neutrophil recruitment and metastasis in mouse models [31].

Clinically, a high neutrophil-to-lymphocyte ratio (NLR) is consistently associated with poorer outcomes in CRC, although it is a nonspecific marker of systemic inflammation and cannot identify its microbial or non-microbial origin [28,32]. Together, these findings suggest that neutrophils may act as intermediaries between microbial signals and tumor-associated inflammation, but the clinical relevance of this axis remains to be defined.

Bacteria also communicate with the tumor microenvironment through bacterial extracellular vesicles (BEVs), nanoscale membrane structures capable of transporting microbial proteins, nucleic acids, and metabolites across epithelial barriers and into distant tissues [33,34]. BEVs derived from *F. nucleatum* activate NF-κB-dependent inflammatory signaling and increase IL-8 production, although their precise contribution to tumor colonization remains incompletely defined [35]. In preclinical settings, *E. coli*-derived outer membrane vesicles induced an interferon-γ-dependent antitumor response after systemic administration in CT26 tumor-bearing mice [36]. Separately, *Lactobacillus plantarum*-derived extracellular vesicles restored 5-fluorouracil sensitivity in resistant CRC cell lines through modulation of PDK2-dependent glucose metabolism [37]. These findings provide proof of principle for BEV-based therapeutic approaches but do not demonstrate clinical activity or establish a role in ICI response. These findings support further investigation of BEVs as diagnostic biomarkers and therapeutic delivery platforms, but their application will require standardized isolation and characterization methods together with robust human safety data [33,34]. The principal microbiota-mediated mechanisms influencing the CRC tumor immune microenvironment and their potential relevance to ICI response are summarized in Figure 1.

## 4. Gut Microbiota and Immune Checkpoint Inhibitor Efficacy

Evidence linking the gut microbiome to ICI efficacy is strongest in melanoma and non-small-cell lung cancer (NSCLC), whereas CRC-specific clinical data remain limited [38]. Although the gut microbiota is anatomically distant from many tumor sites, microbial metabolites and products may enter the circulation and influence systemic dendritic-cell activation, myeloid programming, and T-cell priming, with downstream effects on immune-cell trafficking and effector function. However, the strongest human evidence for these systemic associations comes from melanoma and NSCLC, and causality remains incompletely established. Illustrating a potential preclinical mechanism, Jia et al. showed that indole-3-propionic acid, generated through cooperation between *Lactobacillus johnsonii* and *Clostridium sporogenes*, promoted TCF-1^+^ progenitor-exhausted CD8^+^ T cells and enhanced anti-PD-1 activity in several tumor models, including CRC [39].

In locally advanced rectal cancer, the species-level gut microbiome prediction (SPEED) model showed promising discrimination for pathological complete response to neoadjuvant immunotherapy combined with chemoradiotherapy. The reported AUC was 0.988 in the training cohort and 0.778 in the validation cohort, although the small sample size limits its current clinical applicability [38]. Antibiotic exposure around ICI initiation has also been associated with poorer outcomes across several tumor types, including CRC, but these findings are predominantly observational [38]. Microbiome-based predictors therefore remain investigational and are not ready for routine clinical use.

Microbiome alterations are already present in premalignant colorectal disease. Shotgun metagenomic analysis of fecal samples from healthy controls and patients with advanced adenomas or CRC identified differences in microbial composition and function across the adenoma-carcinoma sequence [6]. *Bifidobacterium animalis*, for example, was depleted in both adenoma and CRC samples, whereas several potentially pathogenic taxa were more abundant in neoplastic disease [6]. Independent studies have likewise reported increased *Fusobacterium* abundance in individuals with colorectal adenomas, supporting the presence of CRC-associated dysbiosis before invasive disease [40]. Subsequent cross-cohort metagenomic analyses identified reproducible CRC-associated microbial signatures across geographically diverse populations, although these studies primarily compared established CRC with tumor-free controls [5].

Beyond their potential use as biomarkers, gut microbial communities are being investigated as therapeutic targets. Early proof-of-concept studies in anti-PD-1-refractory melanoma showed that responder-derived FMT, combined with anti-PD-1 rechallenge, could restore clinical responses in a subset of patients [41,42]. In CRC, the single-arm phase II RENMIN-215 trial reported activity of an FMT-containing regimen in 20 heavily pretreated patients with MSS metastatic disease [43] (Table 1). However, because the intervention combined FMT with tislelizumab and fruquintinib without a control arm, the independent contribution of FMT cannot be determined. More broadly, a meta-analysis of 10 studies including 164 patients with solid tumors reported a pooled ORR of 43% (95% CI, 35–51%), but the evidence was predominantly non-CRC, heterogeneous, and largely derived from small single-arm studies [44]. The pooled estimate showed moderate between-study heterogeneity, further limiting direct comparisons across studies. Differences in donor selection, FMT route, dose and number of administrations, baseline microbiome composition, concomitant anticancer treatment, and prior lines of therapy further limit cross-study comparisons. FMT should therefore remain investigational in CRC pending adequately powered randomized trials with prespecified microbiome and clinical stratification.

## 5. Microbiome-Based Therapeutic Strategies

Microbiome-directed interventions should not be regarded as a single therapeutic class. Their effects depend on the bacterial strain or consortium, formulation, dose, clinical setting, and accompanying anticancer treatment. Precision microbiome therapeutics therefore differ fundamentally from empiric supplementation with commercially available probiotics.

Among candidate NGPs, *Akkermansia muciniphila*, *Bifidobacterium* spp., and *Faecalibacterium prausnitzii* have the strongest mechanistic rationale. As discussed above, these taxa can influence epithelial barrier integrity, antigen presentation, T-cell function, and microbial metabolite production. However, evidence supporting their use as adjuncts to cancer therapy remains predominantly preclinical or observational. In murine CRC models, administration of *A. muciniphila* inhibited tumor growth, enhanced the effects of FOLFOX, and increased intratumoral CD8^+^ T-cell infiltration [46,47]. Its association with anti-PD-1 response has also been demonstrated clinically in NSCLC and renal cell carcinoma, but equivalent prospective evidence in CRC is lacking [48].

Initial human data indicate that oral *A. muciniphila*, including its pasteurized formulation, can be administered safely to metabolically healthy or overweight adults [49,50]. These findings cannot, however, be extrapolated directly to patients with cancer or treatment-related immunosuppression. The safety, optimal dose, and therapeutic efficacy of *A. muciniphila* and other candidate NGPs in oncology therefore require dedicated clinical trials.

Defined bacterial consortia offer a more controlled alternative to single-strain supplementation. In a preclinical study, a defined 11-strain consortium of human-derived commensals induced IFN-γ-producing CD8^+^ T cells and enhanced checkpoint-blockade activity in mice without inducing overt inflammatory pathology [51]. This provides proof of concept for rationally designed multi-strain products, although their clinical utility in CRC remains unproven.

Commercially available probiotics should be distinguished from NGPs because they are not selected for a defined immunological mechanism and vary substantially in strain composition, dose, and formulation. Observational studies in patients receiving ICIs have associated over-the-counter (OTC) probiotic use with poorer treatment outcomes, although residual confounding and the absence of randomized evidence preclude causal conclusions [38]. OTC probiotics should therefore not be recommended specifically for the purpose of improving ICI efficacy outside clinical trials.

Perioperative probiotics and synbiotics represent a clinically more developed application than attempts to enhance ICI efficacy. In a double-blind, placebo-controlled trial, a four-strain probiotic regimen reduced postoperative infectious complications following elective colorectal resection [52]. A meta-analysis of randomized trials likewise suggested benefits for selected infectious outcomes, but found no consistent advantage of probiotics over synbiotics [53]. Marked heterogeneity in strains, doses, treatment schedules, outcome definitions, and study populations prevents regimen-specific clinical recommendations, and long-term oncological benefits have not been established.

Dietary interventions represent another potentially modifiable approach. In observational cohorts of patients receiving ICIs, higher dietary fiber intake has been associated with longer progression-free survival, while early interventional studies suggest that dietary modification may influence intratumoral immune-cell infiltration [38]. However, prospective evidence demonstrating that a high-fiber diet improves ICI efficacy, particularly in CRC, remains limited.

More selective approaches including bacterial extracellular vesicles and bacteriophages targeting bile-acid-modifying bacteria remain preclinical [11,26,33,34]. Their principal advantage is the possibility of modulating defined microbial functions without broadly disrupting the microbiome. Important barriers include manufacturing standardization, endotoxin control, pharmacokinetics, and safety in immunocompromised patients. The evidence base, potential clinical relevance, and current development status of microbiome-directed strategies in CRC are summarized in Table 2.

## 6. Challenges and Future Directions

Most human evidence linking the gut microbiome to CRC biology and immunotherapy outcomes remains observational. Associations identified in individual cohorts may not be reproduced because baseline microbiome composition is influenced by diet, geography, host genetics, antibiotic exposure, concomitant medications, and other clinical factors. Although selected microbiome-based classifiers have achieved reported AUCs of 0.83–0.92, their generalizability across independent populations, sequencing platforms, and analytical pipelines remains uncertain [38]. More broadly, the limited reproducibility of species-level associations suggests that tumor type, treatment regimen, host immune status, and population-specific factors modify the relationship between microbiome composition and ICI response. Future randomized trials of FMT-containing ICI regimens in MSS/pMMR mCRC should include an appropriate control arm and standardized donor screening, donor-selection criteria, route, dose, and number of FMT administrations. Baseline and longitudinal microbiome profiling should be integrated with stratification by prior treatment exposure and molecular and immune characteristics, while safety, objective response, and survival endpoints should be prespecified to distinguish microbiome-related effects from those of concomitant anticancer therapy [43,44].

Methodological heterogeneity represents an additional barrier to reproducibility. Differences in sample collection and storage, DNA extraction, sequencing method, selection of the 16S rRNA variable region, reference databases, and bioinformatic pipelines can generate divergent microbial profiles from otherwise comparable samples. Multicenter studies therefore require harmonized procedures for sample collection, processing, sequencing, and analysis. Dietary intake, antibiotic exposure, proton-pump inhibitors, and other microbiome-modifying medications should also be recorded systematically and incorporated into statistical analyses [38].

Microbial function may ultimately be more informative than the abundance of individual taxa. The immunological effects of SCFAs depend on the specific metabolite, concentration, immune-cell subset, receptor signaling, and tissue environment. Secondary bile acids can similarly exert context-dependent and, in some cases, opposing effects. Consequently, interventions intended simply to increase SCFA production may not be beneficial in every therapeutic setting. In a preclinical intestinal adenocarcinoma-on-chip model, butyrate and propionate reduced the infiltration, cytotoxicity, and cytokine production of anti-ROR1 CAR T cells [25]. These findings should not be extrapolated directly to conventional ICI therapy, but they illustrate the need to combine microbial profiling with metabolomics and detailed immune phenotyping when developing microbiome-directed interventions.

Safety is particularly important in patients with cancer, especially those with impaired mucosal integrity or treatment-related immunosuppression. FMT can transmit pathogenic or multidrug-resistant organisms present in donor material. Transmission of extended-spectrum β-lactamase-producing *Escherichia coli* from a common donor resulted in bloodstream infections in two FMT recipients, one of whom died [57]. This event underscores the need for rigorous donor screening, pathogen surveillance, standardized manufacturing, and careful patient selection. Pasteurized NGPs and other non-viable microbiome-derived products may reduce some risks associated with administering live microorganisms, but their safety in oncological populations must be evaluated directly.

Future biomarker models should integrate microbial functional capacity or circulating and intratumoral metabolite levels with established tumor features, including MSI/MMR status and tumor mutational burden where relevant, immune phenotype, and clinical variables such as treatment line and antibiotic exposure. These models will require prospective validation in geographically diverse cohorts, external testing across analytical platforms, and demonstration of incremental value beyond established biomarkers before clinical implementation. Targeted manipulation of defined microbial functions also represents a promising but early-stage strategy; for example, bacteriophage-mediated depletion of the DCA-producing bacterium *Clostridium scindens* restored CD8^+^ T-cell activity and limited tumor growth in murine CRC models [26].

## 7. Conclusions

The gut microbiota contributes to CRC biology through effects on epithelial integrity, inflammation, DNA damage, and antitumor immunity. However, its influence is not uniform. Individual bacteria and microbial metabolites act through different pathways, and their effects depend on the host, tumor, tissue environment, and treatment setting. The microbiome is therefore one of several factors shaping the immunologically “cold” phenotype of MSS/pMMR CRC, rather than its principal cause.

Clinical evidence is limited. Early findings from FMT-containing combination regimens in MSS metastatic CRC are encouraging, but the contribution of FMT cannot yet be separated from that of the accompanying anticancer agents. Next-generation probiotics and other targeted microbiome approaches also remain investigational. Perioperative probiotics or synbiotics may reduce selected infectious complications after colorectal surgery, although the evidence is heterogeneous across formulations, treatment protocols, and outcome definitions, precluding regimen-specific recommendations. OTC probiotics should not be recommended for the purpose of improving ICI efficacy, and necessary antibiotic treatment should not be withheld.

Before microbiome-based biomarkers or interventions can enter routine CRC care, they require confirmation in adequately powered randomized trials using standardized sampling and analytical methods.

## Figures and Tables

**Figure 1:**
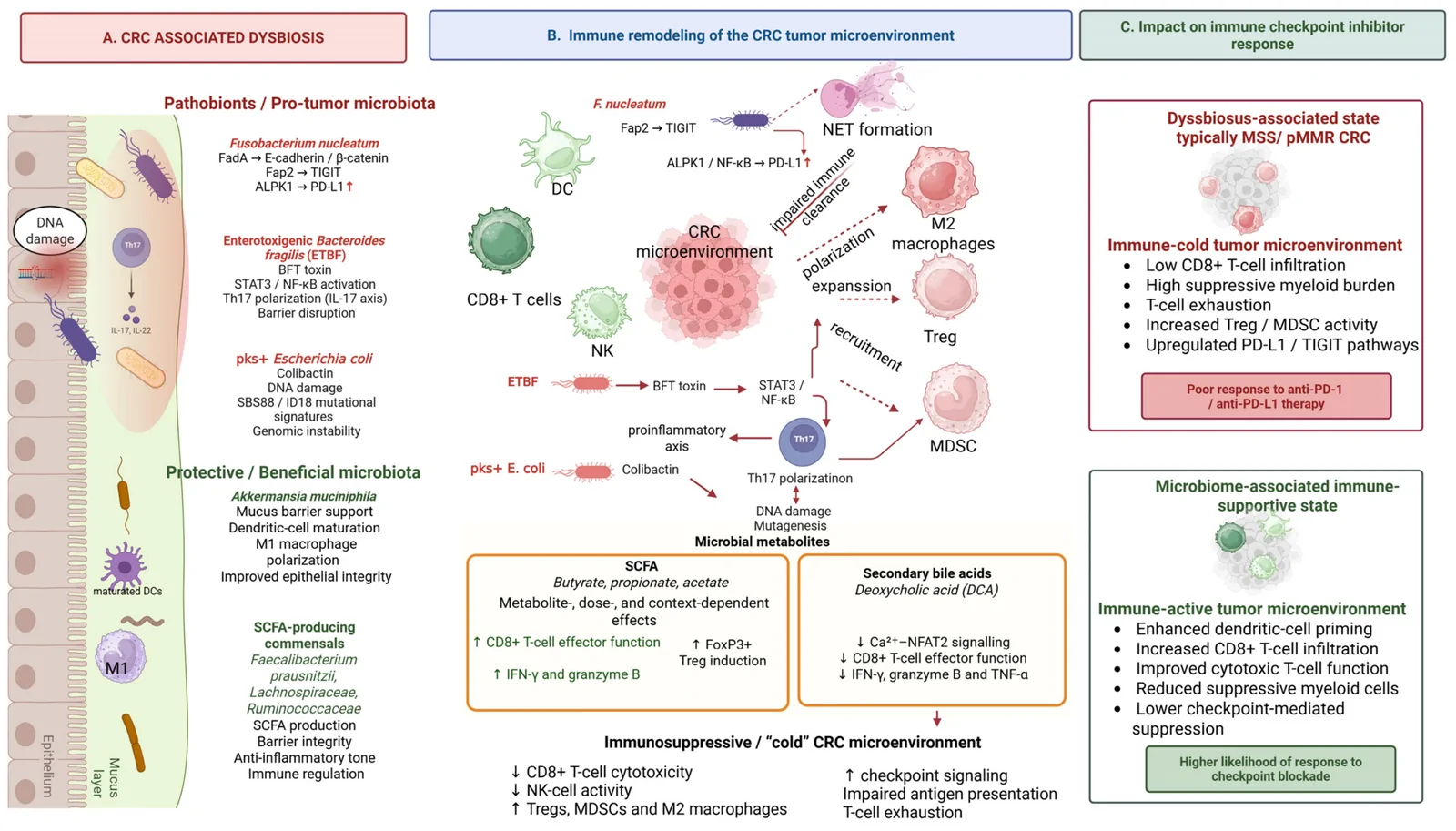
Gut microbiota-immune interactions and their potential relevance to immune checkpoint inhibitor response in colorectal cancer. (**A**) Colorectal cancer-associated dysbiosis is characterized by enrichment of pathobionts, including *Fusobacterium nucleatum*, enterotoxigenic *Bacteroides fragilis*, and pks^+^ *Escherichia coli*, together with alterations in potentially beneficial commensals. These microorganisms can affect epithelial integrity, inflammatory signaling, DNA damage, and immune-cell function through distinct mechanisms. (**B**) Microbial components and metabolites remodel the tumor immune microenvironment by influencing cytotoxic lymphocytes, macrophages, regulatory T cells, myeloid-derived suppressor cells, immune-checkpoint expression, and neutrophil extracellular trap formation. The effects of short-chain fatty acids are metabolite-, dose-, and context-dependent, whereas deoxycholic acid can impair Ca^2+^-NFAT2 signaling and reduce CD8^+^ T-cell effector function. (**C**) These mechanisms may contribute to an immunologically “cold” microenvironment and limited sensitivity to immune checkpoint blockade in MSS/pMMR CRC. Conversely, microbiome-associated immune-supportive states may favor dendritic-cell priming, CD8^+^ T-cell infiltration, and cytotoxic activity. These relationships are context-dependent and should not be interpreted as evidence that microbiome composition independently determines response to immunotherapy. Abbreviations: BFT, *Bacteroides fragilis* toxin; CRC, colorectal cancer; DCA, deoxycholic acid; DC, dendritic cell; ETBF, enterotoxigenic *Bacteroides fragilis*; ICI, immune checkpoint inhibitor; MDSC, myeloid-derived suppressor cell; MSS, microsatellite stable; NET, neutrophil extracellular trap; NFAT2, nuclear factor of activated T cells 2; NK, natural killer; PD-1, programmed cell death protein 1; PD-L1, programmed death-ligand 1; pMMR, proficient mismatch repair; SCFA, short-chain fatty acid; TIGIT, T-cell immunoreceptor with immunoglobulin and ITIM domains. Upward and downward arrows indicate increases and decreases, respectively.

**Table 1:** Clinical studies of FMT-containing regimens relevant to immune checkpoint blockade in colorectal cancer.

Study	Design	Population and Intervention	Main Findings	Main Limitation
Zhao et al., RENMIN-215 [43]	Phase II, single-arm; *n* = 20	Refractory MSS mCRC; FMT + tislelizumab + fruquintinib	ORR 20%; DCR 95%; median PFS 9.6 months; median OS 13.7 months	No control arm; the independent contribution of FMT cannot be determined
Cheng et al. [45]	Case report	pMMR/MSS stage IVB CRC; gut microbiota transplantation + anti-PD-1 + anti-VEGF therapy	Clinical and radiological response accompanied by changes in gut microbial composition	Single patient; multimodal treatment precludes attribution of benefit to microbiota transplantation
Lin et al. [44]	Meta-analysis; 10 studies, *n* = 164	Mixed advanced solid tumors treated with FMT-based microbiome modulation plus ICIs; only one CRC-specific study	Pooled ORR across all tumor types: 43%; MSS mCRC subgroup: 20%	Pooled result is not CRC-specific; small, heterogeneous and predominantly uncontrolled studies

CRC, colorectal cancer; DCR, disease control rate; FMT, fecal microbiota transplantation; ICI, immune checkpoint inhibitor; IVB, stage IVB; mCRC, metastatic colorectal cancer; MSS, microsatellite stable; ORR, objective response rate; OS, overall survival; PD-1, programmed cell death protein 1; PFS, progression-free survival; pMMR, proficient mismatch repair; VEGF, vascular endothelial growth factor.

**Table 2:** Summary of microbiome-based therapeutic approaches in CRC.

Strategy	Evidence Base	Highest Evidence Level	CRC Specificity	Clinical Status/Key Limitation
Pasteurized *A. muciniphila* and bacterial components	CRC mouse models; associations with ICI response mainly in NSCLC and RCC; phase I safety study in adults with overweight or obesity [49,50]	Preclinical CRC evidence; human safety outside oncology	Partly CRC-specific	Investigational; potential barrier and immune effects in experimental models, but CRC efficacy and oncology safety are unestablished.
*Bifidobacterium* spp.	Preclinical ICI models; inosine-A2AR-dependent immune modulation [54]; macrophage and antigen-presenting-cell reprogramming [19]; clinical associations mainly outside CRC	Preclinical; no dedicated CRC clinical trial.	Predominantly extrapolated	Strain-dependent preclinical potential; no dedicated CRC clinical trial.
*Faecalibacterium prausnitzii*	Butyrate production and secretion of the microbial anti-inflammatory molecule (MAM); associations with ICI outcomes mainly in melanoma and other solid tumors [55,56]	Human correlative and preclinical	Predominantly extrapolated	No dedicated therapeutic CRC trial; clinical evidence remains indirect.
Defined 11-strain bacterial consortium	Human-derived commensal consortium induced IFN-γ^+^ CD8^+^ T cells and enhanced antitumor immunity in mouse models [51]	Preclinical	Extrapolated to CRC	Proof of concept for rationally designed consortia; preclinical/early translational development.
Responder-derived FMT	Early-phase melanoma studies; RENMIN-215 in MSS mCRC; meta-analysis of 10 heterogeneous studies reporting a pooled ORR of 43% [41,42,43,44]	Human single-arm study in CRC	CRC-specific, but limited	Experimental; FMT-specific benefit cannot be separated from concomitant therapy, and randomized CRC evidence is lacking.
Perioperative probiotics or synbiotics	Multiple randomized trials and meta-analyses in patients undergoing colorectal surgery, including Kotzampassi et al. [52]	Human RCTs and meta-analyses	CRC surgery-specific	May reduce selected postoperative infections, but heterogeneous formulations and outcomes preclude regimen-specific recommendations.
Non-targeted OTC probiotics during ICI therapy	Observational associations with poorer ICI outcomes, predominantly outside CRC [38]	Human observational	Predominantly extrapolated	No evidence of benefit for ICI efficacy; should not be used specifically to enhance ICI response outside trials.
Bacteriophage-based targeting	Preclinical targeting of pathobionts or metabolite-producing bacteria, including *F. nucleatum* and *C. scindens* [11,26]	Preclinical	Limited CRC-specific evidence	Potentially selective approach, but delivery, resistance, specificity and safety require validation.
Bacterial extracellular vesicles	Antitumor and immunomodulatory effects of selected commensal-derived BEVs in experimental CRC models [33,34]	Preclinical	Partly CRC-specific	Potential cell-free platform; manufacturing, biodistribution, endotoxin-related toxicity and human safety remain unresolved.

A2AR, adenosine A2A receptor; BEV, bacterial extracellular vesicle; CRC, colorectal cancer; FMT, fecal microbiota transplantation; ICI, immune checkpoint inhibitor; IFN, interferon; MAM, microbial anti-inflammatory molecule; mCRC, metastatic colorectal cancer; MSS, microsatellite stable; NSCLC, non-small-cell lung cancer; ORR, objective response rate; OTC, over-the-counter; RCC, renal cell carcinoma; RCT, randomized controlled trial.

## Data Availability

No new data were created or analyzed in this study. Data sharing is not applicable to this article.

## Abbreviations

AUC	area under the curve
BEV	bacterial extracellular vesicle
BFT	bacteroides fragilis toxin
CRC	colorectal cancer
CAR	chimeric antigen receptor
DC	dendritic cell
DCA	deoxycholic acid
DCR	disease control rate
dMMMR	mismatch repair-deficient
ETBF	enterotoxigenic *Bacteroides fragilis*
FMT	fecal microbiota transplantation
ICI	immune checkpoint inhibitor
mCRC	metastatic colorectal cancer
MSI-H	high microsatellite instability
MSS	microsatellite-stable
MDSC	myeloid-derived suppressor cell
NET	neutrophil extracellular trap
NGP	next-generation probiotic
NK	natural killer
NLR	neutrophil-to-lymphocyte ratio
NSCLC	non-small-cell lung cancer
ORR	objective response rate
OS	overall survival
OTC	over-the-counter
PD-1	programmed cell death protein 1
PD-L1	programmed death-ligand 1
pMMR	proficient mismatch repair
PFS	progression-free survival
RCT	randomized controlled trial
SCFA	short-chain fatty acid
TIGIT	T-cell immunoreceptor with immunoglobulin and ITIM domains
TIME	tumor immune microenvironment
